# Brain biomarkers and pre-injury cognition are associated with long-term cognitive outcome in children with traumatic brain injury

**DOI:** 10.1186/s12887-017-0925-6

**Published:** 2017-07-24

**Authors:** Amy A. Wilkinson, Maureen Dennis, Nevena Simic, Margot J. Taylor, Benjamin R. Morgan, Helena Frndova, Karen Choong, Craig Campbell, Douglas Fraser, Vicki Anderson, Anne-Marie Guerguerian, Russell Schachar, Jamie Hutchison, For the Canadian Critical Care Trials Group (CCCTG), The Canadian Critical Care Translational Biology Group (CCCTBG)

**Affiliations:** 1 Department of Psychology, University of Toronto, Toronto, Canada; 2 Program in Neuroscience & Mental Health, The Hospital for Sick Children, Toronto, Canada; 3 Department of Surgery, University of Toronto, Toronto, Canada; 4 Comprehensive Pediatric Epilepsy Program, Hamilton Health Sciences Corporation, Hamilton, Canada; 5 Department of Diagnostic Imaging, The Hospital for Sick Children, Toronto, Canada; 6 Department of Critical Care Medicine, The Hospital for Sick Children, Toronto, Canada; 7 Division of Pediatric Intensive Care, Department of Pediatrics, Children’s Hospital of Hamilton, Hamilton, Canada; 8 Pediatrics, Clinical Neurological Sciences and Epidemiology, Schulich School of Medicine, Western University, London, Canada; 9 Clinical Sciences, Murdoch Children’s Research Institute, Melbourne, Australia; 10 Psychological Sciences and Pediatrics, University of Melbourne, Melbourne, Australia; 11 Department of Psychiatry, The Hospital for Sick Children, Toronto, Canada

**Keywords:** Attention, Executive functions, Traumatic brain injury, Serum biomarkers

## Abstract

**Background:**

Children with traumatic brain injury (TBI) are frequently at risk of long-term impairments of attention and executive functioning but these problems are difficult to predict. Although deficits have been reported to vary with injury severity, age at injury and sex, prognostication of outcome remains imperfect at a patient-specific level. The objective of this proof of principle study was to evaluate a variety of patient variables, along with six brain-specific and inflammatory serum protein biomarkers, as predictors of long-term cognitive outcome following paediatric TBI.

**Method:**

Outcome was assessed in 23 patients via parent-rated questionnaires related to attention deficit hyperactivity disorder (ADHD) and executive functioning, using the Conners 3rd Edition Rating Scales (Conners-3) and Behaviour Rating Inventory of Executive Function (BRIEF) at a mean time since injury of 3.1 years. Partial least squares (PLS) analyses were performed to identify factors measured at the time of injury that were most closely associated with outcome on (1) the Conners-3 and (2) the Behavioural Regulation Index (BRI) and (3) Metacognition Index (MI) of the BRIEF.

**Results:**

Higher levels of neuron specific enolase (NSE) and lower levels of soluble neuron cell adhesion molecule (sNCAM) were associated with higher scores on the inattention, hyperactivity/impulsivity and executive functioning scales of the Conners-3, as well as working memory and initiate scales of the MI from the BRIEF. Higher levels of NSE only were associated with higher scores on the inhibit scale of the BRI.

**Conclusions:**

NSE and sNCAM show promise as reliable, early predictors of long-term attention-related and executive functioning problems following paediatric TBI.

## Background

Following traumatic brain injury (TBI), children and adolescents experience changes in both cognitive and behavioural functioning [1]. These deficits are frequently associated with damage to the frontal and temporal regions of the brain [2]. The frontal lobes are known to have protracted development throughout childhood and adolescence [3], thus are particularly vulnerable to insult, such as TBI, experienced during development. Two cognitive areas that are dependent on intact functioning of frontal networks, and thus frequently reported as impaired following TBI, are executive functioning and attention [4].

Executive functioning is an umbrella term used to describe a variety of abilities allowing purposeful, goal-directed, problem-solving behaviour, including behavioural regulation, planning and organizational skills, and self-monitoring [5]. Deficits in executive functioning have been seen within the first year [6, 7] and up to five [8] and 10 years [4] following childhood TBI. At 10 years post-injury, 26% of those who sustained a moderate TBI and 42% of those who sustained a severe TBI had clinically significant impairments on measures of executive functioning [4]. Deficits in executive functioning have been variously related to injury severity [4, 6–8], age at injury [6] and socioeconomic status (SES) [6].

Behavioural symptoms of attention difficulties are observed as inattention, hyperactivity and impulsivity, which are the primary symptoms associated with attention deficit hyperactivity disorder (ADHD) [9]. These symptoms are over-represented in children with TBI, with 20% of children meeting ADHD criteria prior to the injury [10] and an additional 18–20% of children experiencing de novo attention difficulties by two years post-injury [11, 12]. A relation between injury severity and the development of attention problems has been reported in some studies [11–14], but not others [10, 15, 16]. Thus, standard measures of injury severity do not seem to capture the true biological impact of the trauma. Other associated variables, such as SES, age at injury and sex, are also not reliable predictors of attention difficulties across studies [10, 11, 14–16].

These attention and executive functioning deficits are particularly relevant to children and adolescents as they impact progress in academics and psychosocial development, and may have a negative influence on family functioning [6, 8, 11]. If clinicians were able to identify patients at high risk of long-term deficits in attention and executive functioning, management strategies could be applied in the early stages of recovery to improve functioning and quality of life for the children with TBI and their families.

Due to the poor reliability of clinical factors, recent research in childhood TBI has started to focus on the use of serum biomarkers as predictors of outcome. A few serum biomarkers, such as neuron specific enolase (NSE), S100 calcium binding protein B (S100B), interleukin-6 (IL-6) and interleukin-8 (IL-8), have previously been shown to be associated with global neurological function and cognitive outcomes in children with TBI [17–21]. These studies did not allow for a comprehensive understanding of post-injury cognitive and behavioural deficits that may occur in those who appear to have recovered physically and yet still have serious cognitive sequelae [22].

We conducted a proof of principle study to determine which combination of injury (Glasgow Coma Scale; GCS) and child (age at injury, sex, SES, and pre-injury functioning) variables and six brain-injury and inflammatory serum protein biomarkers relate to long-term outcome in attention and executive functioning, as rated by parents, following childhood TBI. Serum biomarkers S100B, NSE, Il-6 and IL-8 were chosen due to their association with outcome following paediatric TBI. Soluble neuron cell adhesion molecule (sNCAM) and soluble vascular cell adhesion molecule (sVCAM-1) were also measured as potential biomarkers of microvascular injury and inflammation that may be associated with outcome following TBI. This study identifies the characteristics that may be most closely associated with long-term cognitive and behavioural outcome in attention and executive functioning following paediatric TBI. We hypothesized a combination of serum biomarkers [21, 22] will be more strongly associated with long-term outcome measured at least a year and a half post-injury than other child and injury related variables, which have been inconsistently related to cognitive and behavioural outcome in the past [4, 6–8, 10, 11, 14–16].

## Materials and methods

### Participants

**Fig. 1:**
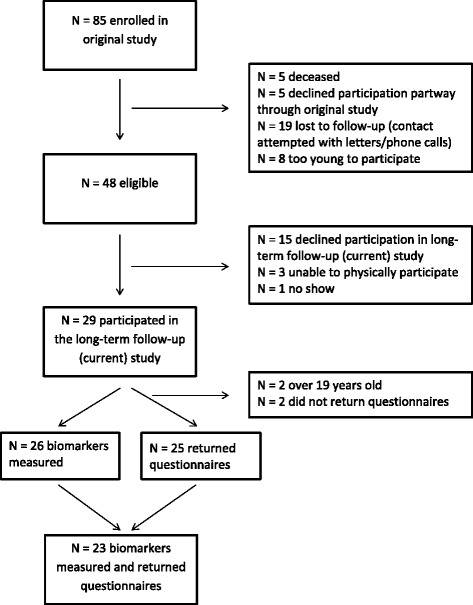
Number of children enrolled in study

Children and adolescents with TBI were recruited from three Ontario children’s hospitals: The Hospital for Sick Children (SickKids; Toronto), Children’s Hospital at London Health Sciences Centre (LHSC; London) and McMaster Children’s Hospital (MCH; Hamilton). Children were recruited from 2009 to 2013 in a prospective observational study, which included a 12 month follow-up time period post-injury. For the present study a subgroup of children were recruited from this convenience sample and asked to return for a follow-up research study from 2012 to 2015. Participants returned to complete the follow-up testing at either SickKids or LHSC. Inclusion criteria for participants were as follows: diagnosis of mild to severe TBI, aged 2.5–17 years old at injury time, parents or guardians were English-speaking and consented to the study. Written informed consent was obtained from participants 18 years old or from parents/guardians of those under 18 years along with assent from the minor participants. Participants were excluded at the time of follow-up if they were over the age of 19 years, as the questionnaires used are not normalized for those over 18.9 years. Recruitment details can be found in Fig. 1. The data were obtained following review by ethics boards at all participating hospitals and in compliance with Canadian National Research Council standards.

### Methods and measures

Using a procedures manual, trained research coordinators collected information on demographics and injury variables. TBI severity was determined by selecting the highest (i.e., the best) of two GCS scores recorded at the scene of the accident and once admitted to hospital. SES was recorded for each participant at the time of follow-up only. As an estimate for SES, parents or guardians were asked to select one of seven categories to indicate total family income (i.e. choices ranged from less than $20,000 to greater than $70,000).

In the acute phase following injury, serum biomarkers were collected and caregivers were administered a questionnaire to assess pre-injury functioning of the patient. Daily blood samples, collected during morning blood work for up to two weeks post-injury, were allowed to clot in a tube with no anticoagulant, centrifuged at 5000 rpm at 4 degrees centigrade (°C) for 10 min, and then separated into 100 μl aliquots. Samples were initially stored at the collecting hospital at −80 °C and then shipped on dry ice and stored at −80 °C at SickKids in a biobank. A laboratory technician, blinded to patient information, completed the biomarker measurements at the Analytical Facility for Bioactive Molecules (SickKids). The following multiplex immunoassays (Millipore-EMD, Billerica, MD, USA) were run on a Luminex 200 using xPonent 3.1.971.0 software (Luminex Corporation, Austin, TX, USA): NSE - MILLIPLEX MAP Human Cancer/Metastasis Biomarker Magnetic Bead Panel – Cancer Multiplex Assay; IL-6 and IL-8 - MILLIPLEX MAP Human Cytokine/Chemokine Magnetic Bead Panel – Immunology Multiple Assay; sVCAM-1 and sNCAM - MILLIPLEX MAP Human Neurodegenerative Disease Magnetic Bead Panel 3 – Neuroscience Multiplex Assay. Also, S100B was measured using Human S100B enzyme linked immunosorbent assay (ELISA) kits. All measurements were conducted using the manufacturers’ instructions.

The highest of the collected levels was determined for five of the six sampled biomarkers for each patient. The highest levels were chosen as opposed to initial levels as serum concentration levels peak at different times for each biomarker and the initial level may not be fully representative of the extent of the injury [19]. The lowest level of sNCAM was selected, as a decrease in this biomarker has been seen following TBI. Blood was drawn for each participant once each day as long as blood was sampled for clinical monitoring. If blood was only drawn once, the serum biomarker levels taken at that time for the patient were used as a proxy for the highest or lowest level.

The Pediatric Injury Functional Outcome Scale (PIFOS) is a brief injury-specific rating scale for children aged 3 to 15 years administered through a caregiver interview by a trained health care provider [23]. It assesses six areas of function: motor skills, daily living skills, communications, social-emotional, cognition and physical changes. It consists of 26 items and uses a four-point scale (0 = no change from preinjury levels; 2 to 4 = increasing need for support and limitations to daily activities). Thus, higher PIFOS scores reflect greater difficulties. The PIFOS was validated by relating it to multiple global and neuropsychological measures, including the Behavior Rating Inventory of Executive Function (BRIEF) [23]. In order to adapt the PIFOS as a measure of pre-injury functioning, the PIFOS questions were rewritten to be posed in the past tense and included the addition of ‘prior to injury’ at the end of each question. A score of 0 was taken to mean no concerns in the area being assessed, while scores of 2 to 4 represented any supports or limitations to daily activities needed pre-injury. The PIFOS was completed by a parent or guardian as a level of ‘baseline’ functioning soon after the child was admitted to hospital with a TBI.

Patients were asked to return for follow-up assessment at least one year following injury. Patients recruited from SickKids and MCH completed follow-up assessment at SickKids, while those recruited from LHSC completed follow-up at LHSC. When patients returned for the long-term follow-up, parents were asked to complete the following questionnaires as per the publishers’ instructions.

The BRIEF – Parent Form is a parent questionnaire designed to assess different aspects of executive functioning behaviours in the home environment in children and adolescents aged 5–18 years. It is divided into the Behavioral Regulation Index (BRI) and Metacognition Index (MI). The BRI is made up of three clinical scales: inhibit, shift and emotional control; while, the MI is made up of five clinical scales: initiate, working memory, plan/organize, organization of materials and monitor [24].

The Conners 3rd Edition Rating Scales (Conners-3) – Parent Form is a parent questionnaire used to assess ADHD and its most common associated problems and disorders in youth aged 6–18 years. The questionnaire consists of six content scales: inattention, hyperactivity/impulsivity, learning problems, executive functioning, peer relations and defiance/aggression [25].

For both the BRIEF and Conners-3, raw scores for each content scale are converted into standardized T-scores, normed for both age and sex. T-scores have a mean of 50 and a standard deviation of 10. Higher T-scores are associated with greater parent-reported concerns, with a T-score of ≥60 considered to be ‘borderline’ and ≥65 considered to be clinically significant [24, 25].

### Statistical analyses

Associations between injury characteristics, serum biomarkers and behavioural measures were analyzed using a partial least squares (PLS) analysis. PLS allows for more accurate predictions than multiple regression derived models, as it can include multiple outcome predictors and has greater stability in handling multicollinearity between variables [26]. PLS identifies patterns between independent and dependent variables. Specifically, the covariance between independent (**X**) and dependent (**Y**) variables is decomposed into components, and these components represent contributions of predictor variables to a pattern of outcome variables. We performed three separate PLS analyses. The predictors were consistent for each analysis and included age at injury, GCS, sex, SES, PIFOS Total score, PIFOS Cognition score, highest levels of S100B, NSE, IL-6, IL-8, sVCAM and lowest levels of sNCAM. These predictors represent individual and injury characteristics and serum biomarkers. The set of outcome variables were different for each analysis. The first analysis looked at all six content scales of the Conners-3, the second looked at the three clinical scales of the BRIEF BRI and the third examined the five clinical scales of the BRIEF MI. All continuous variables for the predictors and outcomes were z-scored, and non-continuous variables (sex and SES) were zero-centred.

The PLS analyses were performed using a combination of MATLAB (Mathworks Inc., Natick MA) and R statistical software. First, using the polycor package in R, a heterogeneous correlation matrix was calculated between each **X** and **Y** data matrix. This method computes Pearson correlations between two continuous variables, polyserial correlations between numeric and discrete variables, and polychoric correlations between two discrete variables. Next, this correlation matrix was decomposed using singular value decomposition (SVD) to obtain orthogonal components, or patterns, which maximize the correlation between predictor and outcome measures. Statistical significance of contributions to each pattern was then computed using bootstrap resampling. The above calculations were performed 5000 times, using random sampling with replacement. On each iteration, an alignment of the eigenvectors (found in the SVD) to the original, non-bootstrapped data was performed. In cases with more than one dependent variable, a Procrustes rotation performed this alignment. Bootstrap ratios were calculated from these distributions, and can be interpreted as a z-score [27]. Significance threshold was set at |Z| > 2.58. The number of components found from SVD equals the number of outcome variables used in the analysis. Significance of a component was determined using an eigenvalue >1 (as found from the SVD). To visualize these patterns, the significant contributors from each analysis were plotted with 95% prediction intervals, as calculated from bootstrap resampling.

We performed a sensitivity analysis to compare included participants from those excluded from the study, independent t-tests, for continuous demographic variables, and Chi-square analyses, for categorical demographic variables, were performed. Independent t-tests were also performed on biomarker levels to compare those included and excluded from this study.

## Results

Twenty-nine of the 85 children and adolescents who were recruited into the original study completed an extended follow-up assessment at more than one year post-injury. Of these participants, 23 had serum biomarkers measured at time of injury and returned completed questionnaires at a long-term follow-up time point, and thus were analyzed in the present study. The patient demographics, injury severity, injury mechanisms and associated injuries are shown in Table 1. Participants included in the follow-up did not differ significantly in terms of demographics and injury characteristics from those in the larger cohort of 85 patients with TBI, who did not participate in this study. However, there were differences between groups for mechanism of injury.

**Table 1:** Demographic variables, injury severity, mechanism of injury, and associated injuries

Characteristic	Full Sample (*n* = 85)	Participants (*n* = 23)
Age at injury in years; mean (SD)	10.54 (4.7) Range: 0.0–17.9	10.95 (3.7) Range: 2.8–15.4
Male; n (%)	64 (75.3)	14 (60.9)
GCS; median (IQR)	10 (9)	9 (9)
Mild; n (%)	30 (35.3)	10 (43.5)
Moderate; n (%)	21 (24.7)	4 (17.4)
Severe; n (%)	34 (40.0)	9 (39.1)
Intubated; n (%)	52 (60.5)	13 (56.5)
PIFOS Total Score	5.86 (9.8)	4.59 (5.8)
PIFOS Cognition Score	2.29 (4.6)	1.50 (2.5)
Mechanism of Injury	n (%)	n (%)
Motor vehicle collision	39 (45.9)	11 (47.8)
Bicycle	14 (16.5)	0 (0)*
Fall	16 (18.8)	5 (21.7)
Sport	7 (8.2)	5 (21.7)*
Other	9 (10.6)	2 (8.7)
CT Findings	n (%)	n (%)
Subdural hematoma	46 (54.1)	11 (47.8)
Epidural hematoma	11 (12.9)	3 (13.0)
Subarachnoid hemorrhage	28 (32.9)	9 (39.1)
Midline shift	13 (15.3)	3 (13.0)
Skull fracture	48 (56.5)	10 (43.5)
Other injuries	n (%)	n (%)
Spine fracture	6 (7.0)	2 (8.7)
Spinal cord injury	3 (3.5)	2 (8.7)
Cardiovascular injury	3 (3.5)	2 (7.1)
Thoracic injury	18 (20.9)	6 (26.1)
Abdominal injury	2 (2.3)	1 (4.3)
Genital-urinal injury	2 (2.3)	1 (4.3)
Major fractures	28 (32.6)	8 (34.8)
Peripheral injury	2 (2.3)	0 (0.0)

*SD* standard deviation, *IQR* interquartile range. Independent t-tests, for continuous demographic variables, and Chi-square analyses, for categorical demographic variables, were performed on those included and excluded from the study. *p < .05

**Fig. 2:**
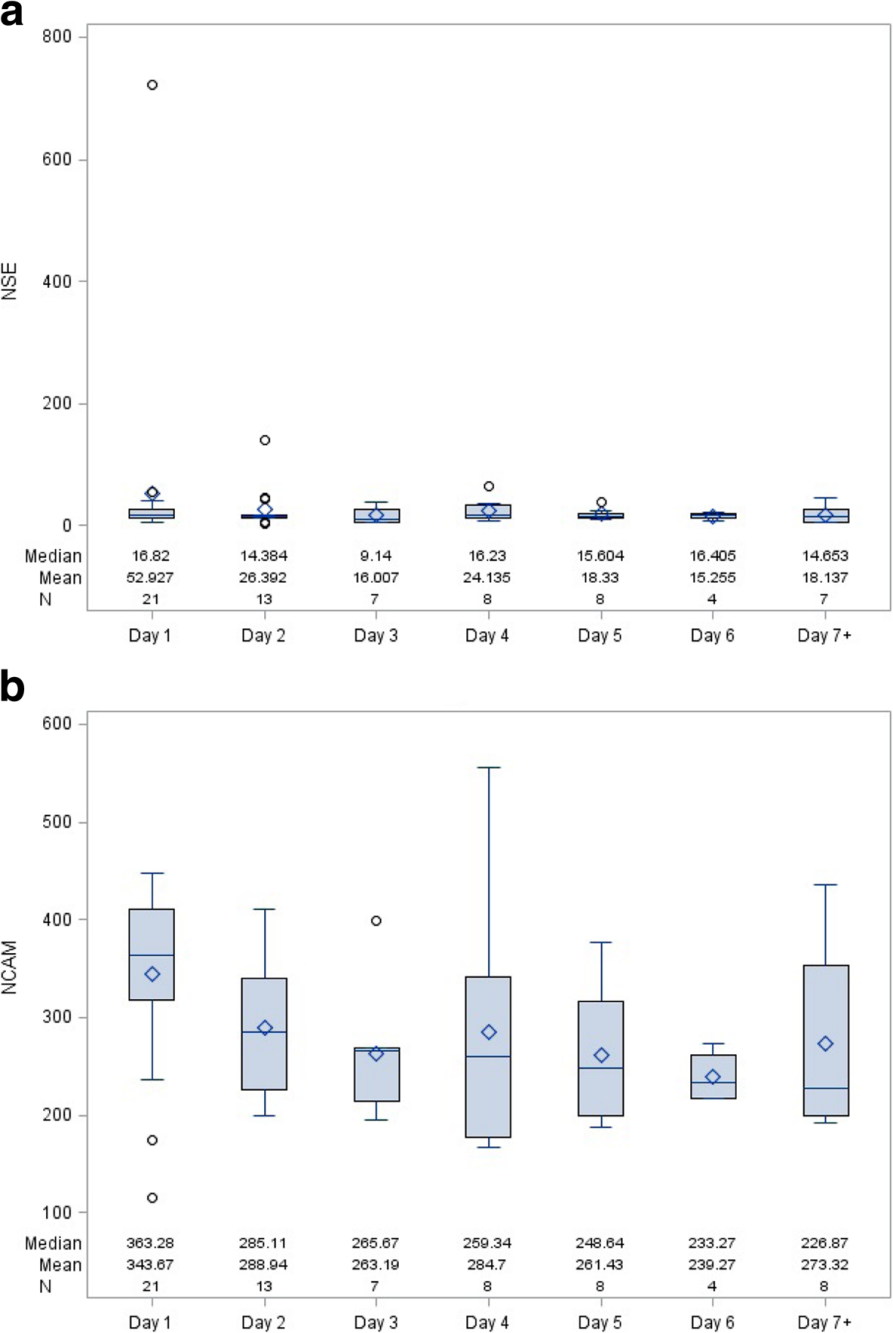
Change in NSE and sNCAM over time following injury. Legend: The distributions of NSE (**a**) and sNCAM (**b**) for each time point are represented by schematic boxplots. The *box* represents the interquartile range (IQR; edges are 25% and 75%), the *line* through the middle of each box represents the median and the *diamond* represents the mean. The *whiskers* extending from the box represent the most extreme points in the group that lie within the fences. The *upper fence* is defined as the third quartile plus 1.5 times the IQR and the *lower fence* is defined as the first quartile minus 1.5 times the IQR. The *circles* represent outliers, which fall outside of the fences

A summary of serum biomarker levels and the time they were collected can be found in Table 2. Seven of the 23 participants had only one blood draw. The first blood draw for these seven participants took place at a mean of 16.51 h following injury. Figure 2 depicts the change in serum levels of NSE (Fig. 2a) and sNCAM (Fig. 2b) over days following injury. Mean T-scores for the 23 participants included in the long-term follow-up portion of the study for the eight clinical scale scores of the BRIEF can be seen in Table 3 and the six content scales of the Conners-3 can be seen in Table 4. Tables 3 and 4 also report the percentage of participants with borderline or clinically significant T-scores on each scale. Children were seen at follow-up between the ages 6.0 and 18.8 years [Mean age = 14.1 years, Standard Deviation (SD) = 4.0 years] and at 1.5 to 5.7 years (Mean time = 3.1 years, SD = 1.1 years) post-injury.

**Table 2:** Mean (SD) highest biomarker levels and the mean (SD) time the highest levels were sampled

	Full Sample (*n* = 76)		Participants (*n* = 23)	
# of blood draws	3.3 (2.4) Range: 1–12		3.0 (1.9) Range: 1–7	
	Highest Level	Time Sampled (Hours)	Highest Level	Time Sampled (Hours)
S100B (pg/ml)	333.9 (512.2) Range: 19.1–3083.4	25.5 (34.3) Range: 0.7–177.7	500.7 (770.6) Range: 22.7–3083.4	19.9 (36.6) Range: 0.7–177.7
NSE (ng/ml)	48.1 (94.8) Range: 4.1–722.2	42.6 (52.1) Range: 0.7–219.8	54.7 (146.3) Range: 4.1–722.2	48.7 (60.3) Range: 1.1–219.8
IL-6 (pg/ml)	143.3 (322.0) Range: 3.7–2471.9	34.5 (45.3) Range: 1.3–287.8	115.3 (194.5) Range: 3.7–745.5	45.8 (63.5) Range: 1.4–287.8
IL-8 (pg/ml)	79.5 (156.4) Range: 4.4–1056.9	36.6 (44.6) Range: 1.3–177.7	53.2 (70.5) Range: 5.8–328.3	35.3 (48.0) Range: 1.4–177.7
sVCAM-1 (ng/ml)	1088.8 (386.1) Range: 601.5–2890.9	45.2 (54.3) Range: 0.7–219.8	1091.3 (456.8) Range: 601.5–2773.2	50.1 (59.1) Range: 1.1–219.8
sNCAM (ng/ml)^a^	280.3 (105.0) Range: 10.1–583.8	55.9 (50.5) Range: 1.3–219.8	284.8 (96.4) Range: 115.3–447.5	52.2 (51.6) Range: 1.4–219.8

The full names of each biomarker and their abbreviations can be found in the methods section of the manuscript. ng/ml = nanograms per millilitre; pg/ml = picograms per millilitre. No significant differences were found on independent t-tests performed on biomarker levels for those included in this study and those excluded. ^a^Mean highest levels of biomarkers were calculated for all except sNCAM, for which the lowest level was used

**Table 3:** Participant results on the clinical scales of the BRIEF

BRIEF Scales	T Scores; mean (SD)	n (%) with T-Scores ≥60	n (%) with T-Scores ≥65
Inhibit	57.09 (15.1)	8 (34.8)	5 (21.7)
Shift	59.78 (15.0)	12 (53.2)	8 (34.8)
Emotional Control	55.78 (12.9)	8 (34.8)	5 (21.7)
Behavioural Regulation Index (BRI)	58.35 (15.1)	9 (39.1)	6 (26.1)
Initiate	56.87 (14.6)	9 (39.1)	7 (30.4)
Working Memory	60.30 (15.6)	9 (39.1)	8 (34.8)
Plan/Organize	59.96 (13.6)	9 (39.1)	7 (30.4)
Organization of Materials	53.61 (11.1)	7 (30.4)	5 (21.7)
Monitor	56.91 (12.6)	8 (34.8)	7 (30.4)
Metacognition Index (MI)	59.04 (13.4)	9 (39.1)	8 (34.8)
Global Executive Composite (GEC)	59.35 (14.8)	10 (43.5)	9 (39.1)

The means and standard deviations of the T-Scores of the BRIEF clinical scales. The number and percentage of the 23 participants with clinically elevated symptoms on each scale are also presented

**Table 4:** Participant results on the content scales of the Conners-3

Conners-3 Content Scales	T Scores; mean (SD)	n (%) with T-Scores ≥60	n (%) with T-Scores ≥65
Inattention	63.09 (15.6)	12 (53.2)	10 (43.5)
Hyperactivity/Impulsivity	61.04 (16.0)	10 (43.5)	9 (39.1)
Learning Problems	58.70 (11.9)	11 (47.8)	9 (39.1)
Executive Functioning	59.73 (13.5)	10 (43.5)	8 (34.8)
Defiance/Aggression	57.74 (15.8)	7 (30.4)	7 (30.4)
Peer Relations	57.70 (15.7)	9 (39.1)	6 (26.1)

The means and standard deviations of the T-Scores of the Conners-3 content scales. The number and percentage of the 23 participants with clinically elevated symptoms on each scale are also presented

### Conners-3

**Fig. 3:**
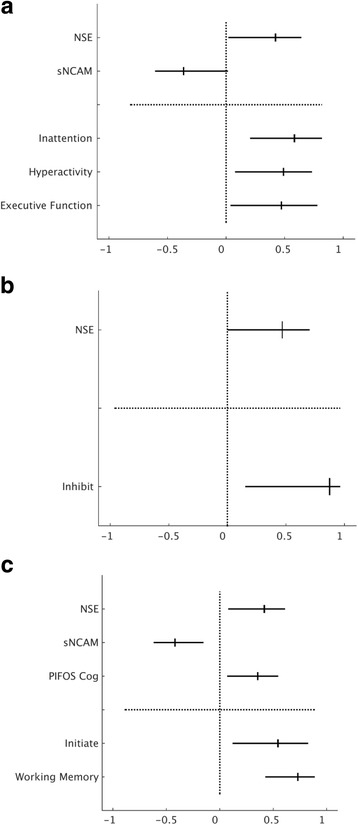
PLS analyses results for Conners-3 content scores and the BRIEF BRI and BRIEF MI clinical scales. Legend: The significant contributions of the independent (*above dotted line*) and dependent (*below dotted line*) variables to the first component for (**a**) the Conners-3 content scales (**b**) the BRIEF BRI clinical scales and (**c**) the BRIEF MI clinical scales for the 23 TBI patients. The *lines* represent the 95% prediction intervals, and the ticks on each line represent the median bootstrapped contribution of each variable. Significant contributions to the component were determined using a bootstrap ratio (z-score) of >2.58

The first PLS analysis revealed one significant component with an eigenvalue of 1.7, which accounted for 75% of the total variance. Significant predictor (**X**) and outcome (**Y**) latent variables were determined by their bootstrap ratios, which can be seen in Table 5. The pattern of association between these variables revealed that higher levels of NSE and lower levels of sNCAM were associated with higher T-scores for inattention, hyperactivity/impulsivity and executive functioning, as shown in Fig. 3a. The other predictors did not significantly contribute to this pattern of outcome measures. Learning problems, aggression and peer relations were not significantly associated with any pattern of independent measures.

**Table 5:** Bootstrap ratios of predictors and outcomes for the three Partial Least Square analyses

Variables	PLS 1	PLS 2	PLS 3
Age at Injury	−0.02	−1.32	−1.86
Sex	−2.32	−2.22	−0.94
GCS	0.67	1.54	0.42
SES	−1.90	−0.61	−1.23
PIFOS Total Score	−0.52	−0.25	1.53
PIFOS Cognition Score	0.39	0.60	3.09*
S100B	1.76	1.40	−0.31
NSE	3.20*	3.17*	3.98*
IL-6	0.78	−0.02	0.62
IL-8	0.43	−0.01	0.97
sVCAM	2.10	1.77	2.20
sNCAM	−2.81*	−2.04	−4.19*
Conners-3 Content Scales			
Inattention	4.25*	-	-
Hyperactivity/Impulsivity	3.23*	-	-
Executive Functioning	3.09*	-	-
Learning Problems	−0.01	-	-
Defiance/Aggression	0.06	-	-
Peer Relations	−0.13	-	-
BRIEF BRI Scales			
Inhibit	-	3.30*	-
Shift	-	0.06	-
Emotional Control	-	1.92	-
BRIEF MI Scales			
Initiate	-	-	3.91*
Working Memory	-	-	6.72*
Plan/Organize	-	-	0.94
Organization of Materials	-	-	−1.04
Monitor	-	-	0.52

The bootstrap ratios are presented for the three Partial Least Squares (PLS) analyses conducted. The bootstrap ratios (z-scores) for the predictor variables are presented in the top half of the table, while the bootstrap ratios for the outcome variables are presented in the bottom half of the table. *Signifies significant differences between the latent variable and the null hypothesis

### BRIEF BRI

The second PLS analysis revealed one significant component with an eigenvalue of 1.7, which accounted for 95% of the total variance. For this component, higher levels of NSE were associated with higher T-scores on the inhibit scale. This relationship can be seen in Fig. 3b, and the bootstrap ratios can be seen in Table 5. No other predictors were significantly associated with outcome.

### BRIEF MI

The final PLS analysis revealed one significant component with an eigenvalue of 2.1, which accounted for 89% of the total variance. The pattern of results among these variables revealed higher levels of NSE, lower levels of sNCAM and a higher PIFOS Cognition score to be associated with higher T-scores on the initiate and working memory scales (see Fig. 3c). Bootstrap ratios for the MI predictors can also be seen in Table 5. The other predictors were not significantly associated with MI outcome.

## Discussion

We identified associations between time of injury variables and cognitive and behavioural outcomes as measured by parent questionnaires. PLS analyses revealed similar patterns of associations between the predictors and all three sets of outcome variables. In a sample of 23 children who had experienced a TBI and were seen at follow-up an average of 3.1 years post-injury, we found that the combination of highest levels of NSE and the lowest measured levels of sNCAM were associated with greater difficulties with inattention, hyperactivity/impulsivity and executive functioning as measured by the Conners-3. We also found that a combination of these two biomarkers combined with a high PIFOS Cognition score was associated with the initiate and working memory scales of the BRIEF MI. The highest levels of NSE were also associated with the inhibit scale of the BRIEF BRI. This profile of predictor variables measured at the time of injury reflect those who are at risk for these specific aspects of attention and executive functioning issues following childhood TBI.

The use of PLS is well suited for this study, as this technique allows the evaluation of large numbers of variables with high multicollinearity, in a relatively small sample and provides insight into the multivariate relations among predictors and outcome variables [26]. In addition to the associations we found between serum biomarkers and outcome, we found that not all pre-injury risk factors and injury characteristics were predictive of cognitive and behavioural outcomes. This is an important consideration as the variables included in this study are routinely used when studying outcome. Injury severity (as measured by GCS), age at injury, SES and sex have been related to cognitive outcome in various studies [1, 4, 6–8, 10, 11, 14–16]. Here, none of those variables were significantly associated; instead we showed a set of specific variables measured at the time of injury were related to long-term attention and executive functioning outcome.

Predicting individual outcomes following TBI based upon pre-injury variables and injury characteristics is an important undertaking. To our knowledge, this is the first study to report on serum biomarkers and long-term cognitive outcomes. Interestingly, the same two biomarkers were consistently part of the clinical profiles associated with outcome. Serum NSE was a significant predictor in all three PLS analyses and serum sNCAM was a significant predictor in two of the analyses. It has been hypothesized that a combination of serum biomarkers or a combination of biomarkers and injury severity would most likely be the best predictors of outcome [21]. NSE has been shown to relate to global outcome using the Glasgow Outcome Scale (a five point scale assessing physical disability) [18–20], but sNCAM has not been previously studied in relation to outcome following paediatric TBI. In a related study, we investigated nine serum biomarkers and their relations to the inattention content scale of the Conners-3 measured 12 months after TBI. While sNCAM was found to be a moderate predictor of inattention at 12 months post-injury, we found the combination of NSE with pre-injury estimates of inattention was the strongest predictor of inattention at 12 months post-injury [28]. The present study has also shown a relation between NSE and sNCAM with three of the Conners-3 content scales at a longer follow-up time, including inattention.

The two content scales of the Conners-3, inattention and hyperactivity/impulsivity, that were part of the outcome profile of the first PLS analysis are the primary symptoms of ADHD [9, 25], which we know is frequently seen in children following TBI [10–12]. These ADHD symptoms have been seen in the chronic stages following injury in previous studies [9, 14], and we have now shown a relation with these symptoms measured in the chronic stages following injury and serum biomarkers measured in the acute period following injury. The executive functioning content scale from the Conners-3 was also part of the outcome profile found in the first PLS analysis. The BRIEF allowed for a more in-depth evaluation of executive functioning outcome in the second and third PLS analyses. To our knowledge, this is the first study to report the relations between serum biomarkers and parent-reported executive functioning following TBI, although other investigators have found long-term deficits in executive functioning post TBI [4, 7, 8]. These studies reported significant declines on the two index scores and the composite score of the BRIEF in children with TBI, but Sesma and colleagues [7] reported significant differences in all of the clinical scales except organization of materials, between children with TBI at a year following injury when compared to both baseline scores and a control group. Working memory was the only clinical scale of the BRIEF that was consistently significantly different following TBI regardless of the severity of TBI or time over the first year following injury. In this current study, working memory was one of the three clinical scales of the BRIEF significantly related to predictors in the two PLS models. A variety of executive functions are evidently impaired following TBI.

The significant association between scales from both questionnaires and serum biomarkers NSE and sNCAM suggests that these serum biomarkers reflect significant brain injury that has long-term sequelae. NSE is a glycolytic enzyme found primarily in the cytoplasm of neurons and released into extracellular space as a result of neuron damage [29]. sNCAM is a binding glycoprotein expressed on the surface of neurons which helps promote neurite outgrowth and is passively released upon cell destruction [30]. Future studies should further characterize the predictive power of these two serum biomarkers in predicting cognitive outcome following paediatric and adult TBI.

For the current study, we did not have pre-injury Conners-3 or BRIEF questionnaires available on all of our participants to study the impact of pre-injury functioning on outcome. We did, however, have the PIFOS as a proxy of pre-injury functioning. The PIFOS Cognition score included questions on judgement/safety, memory, attention, speed of processing, academic placement and executive function, and specifically inquires about impulses, planning and organizing activities and problem solving [23]. Many of these questions tap into the indices of the BRIEF and the PIFOS was originally validated by showing relations with the BRIEF [23]. The questions that make up the PIFOS Cognition score appear to be measuring comparable functions to the clinical scales of the MI rather than the BRI. Thus, this could explain why the predictor profile for the MI outcome included the serum biomarkers as well as the PIFOS Cognition score. This study was designed prior to 2009, and thus prior to the publication of the current National Institute of Health (NIH) TBI common data elements (CDEs) [31]. In accordance with these recommendations, we are currently also collecting the GOS-Extended Pediatric Revision (GOS-E Peds) and other NIH TBI CDEs in ongoing studies.

Parent questionnaires provide ecologically valid assessments of a range of children’s behaviour [8]. The investigation of the relations between serum biomarkers and cognition through neuropsychological measures would allow, however, greater insight into these deficits and would be the obvious next step in the research of serum biomarkers and long-term cognitive sequelae of TBI. Cognitive interventions have not been widely researched in childhood TBI, associations between serum biomarkers and neurocognitive outcome could contribute to planning for early interventions during the acute time period following TBI. Some early studies have shown improvements on the BRIEF and measures of attention and executive functioning following cognitive intervention programs including the Attention Improvement and Management program [32] and a family-centred, online-counselor-assisted program intervention [33, 34].

This proof of principle revealed promising results, but before clinical decision making can be directed the results need to be replicated in a prospective study with a larger sample size. We acknowledge that we were limited by the number of blood sample draws available for each patient, with some patients only having one blood sample drawn, in particular those with mild TBI. In the current study, we required informed consent prior to collecting blood samples, and this may have led to missing the highest serum levels for biomarkers that peak early, such as S100B. In subsequent studies, Research Ethics Boards at hospitals in Canada and Australia have provided permission to use a deferred consent model, allowing the collection of daily blood samples up to 48 h prior to obtaining informed consent.

Additionally, further understanding of these serum biomarkers in both injured and non-injured children is needed. Collecting medically unnecessary blood samples from typically developing controls is a difficult task in the paediatric population, both from a participant and a Research Ethics Board standpoint. One previous study found no significant difference in NSE and S100B levels between two control groups recruited from those undergoing routine blood work or with isolated fractures, but did see significant differences in biomarker levels between these controls and those with head injury [35]. Some of the biomarkers that we investigated in this study are released as a result of injuries to other parts of the body, as outlined in Table 1. Thus, another excellent control group could be those undergoing care for serious injuries without head injury. Future studies should carefully consider the addition of injured and non-injured control groups, as well as the impact of activity to the serum biomarker levels of the patients prior to incurring the injury.

## Conclusions

Following TBI, children are at risk of persistent attention and executive functioning problems. In this proof of principle study, we showed a relation between the serum biomarkers, NSE and sNCAM, with long-term outcome on the Conners-3 and BRIEF. For the MI from the BRIEF, this relationship included a combination of these biomarkers with the PIFOS Cognition score, while the BRI from the BRIEF was only associated with NSE. When validated in future research studies, these associations could improve clinical decisions, prediction of long-term outcome and planning during the acute time period following TBI.

## Abbreviations

°C: Degrees centigrade
ADHD: Attention deficit hyperactivity disorder
BRI: Behavioral Regulation Index
BRIEF: Behavior Rating Inventory of Executive Function
Conners-3: Conners 3rd Edition Rating Scales
ELISA: Enzyme linked immunosorbent assay
GCS: Glasgow Coma Scale
SickKids: The Hospital for Sick Children
IL-6: Interleukin-6
IL-8: Interleukin-8
LHSC: Children’s hHospital at London Health Sciences Centre
MCH: McMaster Children’s Hospital
MI: Metacognition Index
NSE: Neuron specific enolase
PIFOS: Paediatric Injury Functional Outcome Scale
PLS: Partial least squares
S100B: S100 calcium binding protein B
SD: Standard deviation
SES: Socioeconomic status
sNCAM: Soluble neuron cell adhesion molecule
sVCAM-1: Soluble vascular cell adhesion molecule
SVD: Singular value decomposition
TBI: Traumatic brain injury
